# Dissecting the genetic basis of fruiting efficiency for genetic enhancement of harvest index, grain number, and yield in wheat

**DOI:** 10.1186/s12870-025-06072-1

**Published:** 2025-01-24

**Authors:** Dipendra Shahi, Jia Guo, Md Ali Babar, Sumit Pradhan, Muhsin AVCI, Jordan McBreen, Zhao Liu, Guihua Bai, Paul St. Amand, Amy Bernardo, Matthew Reynolds, Gemma Molero, Sivakumar Sukumaran, John Foulkes, Jahangir Khan

**Affiliations:** 1 School of Plant, Environmental and Soil Sciences, Louisiana State Agricultural Center, Louisiana State University, Baton Rouge, LA 70803 USA; 2 Inari Agriculture, 1281 Win Hentschel Blvd w1108, West Lafayette, IN 47906 USA; 3 Department of Agronomy, University of Florida, 3105 McCarty Hall B, Gainesville, FL 32611 USA; 4 Department of Agronomy, Kansas State University, Manhattan, KS 66506 USA; 5 USDA-ARS Hard Winter Wheat Genetics Research Unit, Manhattan, KS 66506 USA; 6 International Maize and Wheat Improvement Center (CIMMYT), Carretera México-Veracruz, Km. 45, El Batán, Texcoco, 56237 México; 7 Queensland Alliance for Agriculture and Food Innovation (QAAFI), The University of Queensland, Hermitage Research Facility, Warwick, Queensland 4370 Australia; 8 Present Address: Department of Plant & Environmental Sciences & Advanced Plant Technology Program, Clemson University, Clemson, SC 29634 USA; 9 Division of Plant and Crop Sciences, School of Biosciences, University of Nottingham, Leicestershire, LE12 5RD UK; 10 PARC-Balochistan Agricultural Research and Development Center, Quetta, 87300 Pakistan

**Keywords:** Fruiting efficiency, Harvest index, KASP marker, GWAS, Marker-trait association

## Abstract

**Background:**

Grain number (GN) is one of the key yield contributing factors in modern wheat (*Triticum aestivum*) varieties. Fruiting efficiency (FE) is a key trait for increasing GN by making more spike assimilates available to reproductive structures. Thousand grain weight (TGW) is also an important component of grain yield. To understand the genetic architecture of FE and TGW, we performed a genome-wide association study (GWAS) in a panel of 236 US soft facultative wheats that were phenotyped in three experiments at two locations in Florida and genotyped with 20,706 single nucleotide polymorphisms (SNPs) generated from genotyping-by-sequencing (GBS).

**Results:**

FE showed significant positive associations with GN, grain yield (GY), and harvest index (HI). Likewise, TGW mostly had a positive correlation with GY and HI, but a negative correlation with GN. Eighteen marker-trait associations (MTAs) for FE and TGW were identified on 11 chromosomes, with nine MTAs within genes. Several MTAs associated with other traits were found within genes with different biological and metabolic functions including nuclear pore complex protein, F-box protein, oligopeptide transporter, and glycoside vacuolar protein. Two KASP markers showed significant mean differences for FE and TGW traits in a validation population.

**Conclusions:**

KASP marker development and validation demonstrated the utility of these markers for improving FE and TGW in breeding programs. The results suggest that optimizing intra-spike partitioning and utilizing marker-assisted selection (MAS) can enhance GY and HI.

**Supplementary Information:**

The online version contains supplementary material available at 10.1186/s12870-025-06072-1.

## Background

Wheat is one of the most widely grown crops worldwide in terms of both area and quantity of production [1]. It is a critical component of global food security, fulfilling approximately 20% of the protein and calorie demand of the world population [2, 3]. At present, the average genetic gain in wheat is lower than 1% per annum; however, 2% of annual yield gain is required [4] to meet the future demand of a 9.5 billion population by 2050 [5]. As cultivable land is already declining in many areas due to soil erosion and degradation, it is crucial to improve the genetic yield potential of crops if we are to avoid further loss of natural ecosystems [6, 7]. A better understanding of the genetic relationship between yield and associated traits may facilitate further breakthroughs in developing high-yielding wheat varieties.

Grain number per unit area (GN) is one of the important determinants of grain yield in wheat [5, 8, 9]. Substantial improvement of GN is essential for achieving large genetic gains in wheat yield as grain filling is limited mainly by the sink capacity in modern wheat cultivars under optimal conditions [5, 10]. Therefore, exploring the physiology and genetics involved in GN determination is necessary for grain yield improvement in wheat. GN has a positive relationship with spike partitioning index (SPI) and can be improved by selecting for higher spike dry weight or SPI [5, 7, 11, 12]. A higher proportion of biomass to the spike during anthesis, which is largley due to reduced height in the past, has resulted in the survival of more fertile florets and greater GN [7, 13, 14]. As the modern varieties are mostly within the optimum stature (0.7–1 m) [15, 16], new ways to improve the grain number need to be studied.

In addition to SPI driving greater spike biomass at anthesis, GN may vary among genotypes due to another important partitioning trait, namely fruiting efficiency (FE). FE is the ratio of the grain number at maturity and spike dry weight at anthesis and can be used as an alternative way to explain GN determination. FE refers to the efficiency of a plant in converting the resources that are allocated for growing spikes before anthesis into grains [7, 17, 18]. Increased allocation of assimilates during spike growth to reproductive structures is associated with increased survival of distal florets and grain formation before anthesis [7, 12, 17], and with decreased allocation to structural components of a spike (rachis, glumes, etc.). Therefore, improved FE is a result of efficient intra-spike partitioning through which assimilates are diverted towards florets rather than spike structural components [7, 19]. Wide genetic variation for intra-spike partitioning traits such as glume partitioning index (GPI), lemma partitioning index (LmPI), and palea partitioning index (PPI) has been reported [19, 20]. Although more attention has been given to SPI for effectively increasing harvest index (HI) in wheat, FE could be employed as a complementary trait for improving GN and HI [7, 9, 21–24]. A positive association between FE and GN has been reported in many studies [8, 9, 25]. Due to a wide range of variation among genotypes [7, 8, 18, 24, 26], along with responsiveness to selection, FE is a novel physiological trait with huge potential for improving GN, grain yield (GY), and harvest index (HI). As we examine FE and its influence on GN, it is relevant to consider a possible trade-off between SPI and FE [8, 26] because some studies have shown a negative relationship between these traits [7, 26, 27] although it could not be confirmed in some other studies [7, 8]. However, wheat cultivars that combined both high FE and SPI have been reported [19, 20]. Likewise, some studies reported a negative relationship between FE and grain weight (GW) [16, 19, 28], but they were unrelated in other studies [22]. Improvement in GN through FE would be discouraged if GW is highly reduced due to the trade-off between FE and GW [7, 16]. Hence, it is important to study the possible trade-off between FE and GW in wheat. It should also be mentioned that measuring FE is a time-consuming task that requires data collection at two stages: measuring spike dry weight at anthesis and counting grain number at maturity [7, 24]. FE measurement is also a destructive process, both of which make it impractical to be applied in progeny selection [7, 29]. Identifying genetic loci controlling FE and associated markers will make it possible to select FE through marker-assisted selection (MAS) to improve HI and GY in breeding programs.

Genome-wide association study (GWAS) is one of the most popular methods for deciphering genetic architectures of complex traits in plants [30]. With advancements in next-generation sequencing (NGS) technologies, low-cost and high-density genome-wide single nucleotide polymorphisms (SNPs) make it possible for most breeding programs to use the genomic tools in routine breeding selection to accelerate crop improvement [30, 31]. To date, only limited research on understanding the genetic basis of FE in wheat has been reported. The purposes of this study are to (i) evaluate associations between FE and HI, GY and GN in a panel of US soft wheat, (ii) study dry matter partitioning amongst spike structural components in a small subset of accessions from the panel, (iii) use GWAS to identify QTLs and molecular markers associated with FE, and (iv) validate KASP markers developed from the QTL information in a new population.

## Results

### Genetic variation and heritability

**Fig. 1:**
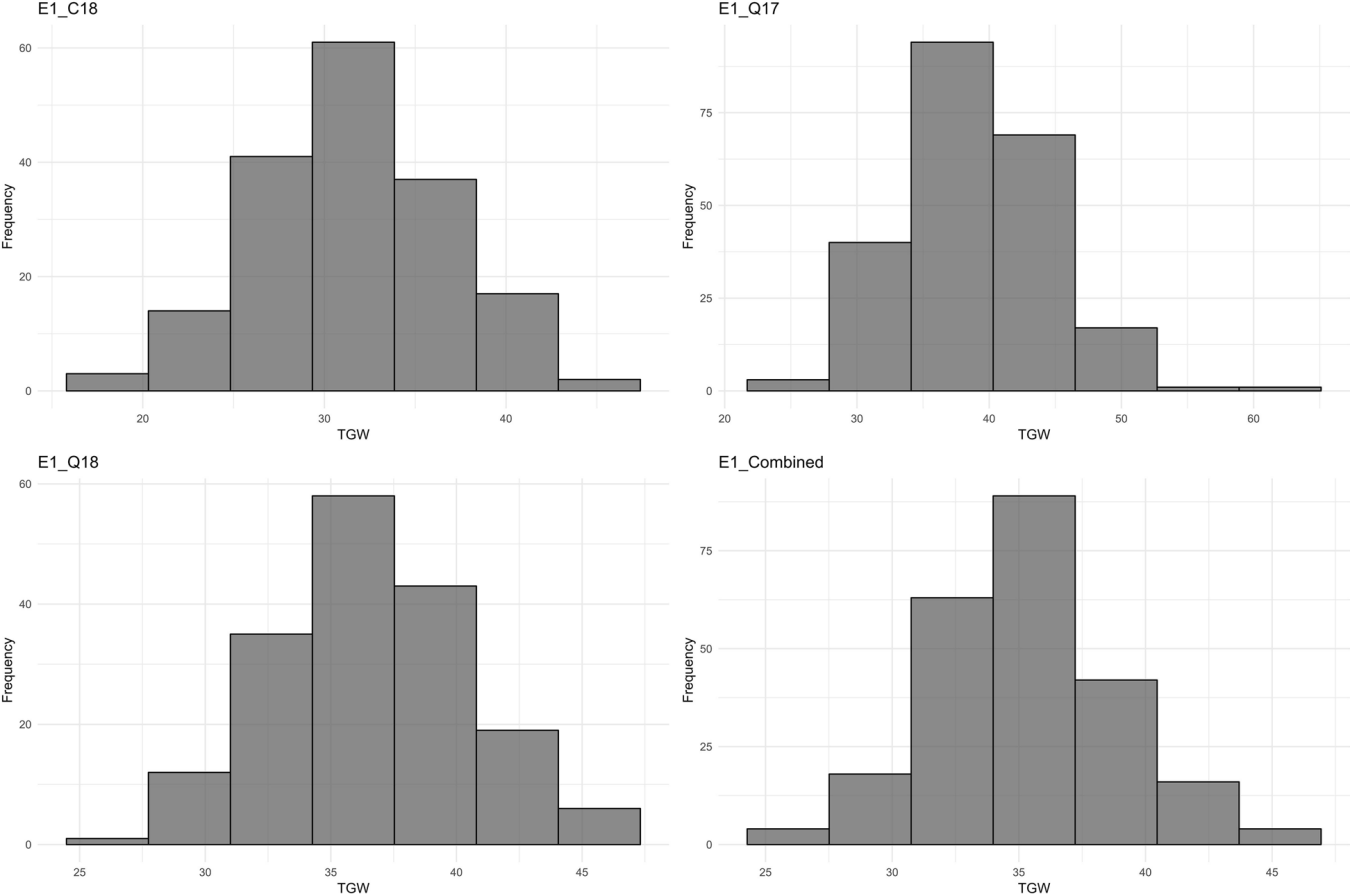
Distribution of TGW, thousand grain weight values. E1_C18, E1_Q17, E1_Q18, and E1_Combined refers to Citra 2018, Quincy 2017, Quincy 2018, and combined dataset for experiment I

A combined analysis of variance (ANOVA) displayed significant genotypic variations for all the measured traits (Table S1) in Experiment I. Significant effects of environments and genotype-by-environment interactions on FE and TGW were observed. The distribution of TGW values from different environments are presented in Fig. 1. The mean phenotypic values ranged from 33.69 (E1_Q17) to 49.56 (E1_Q18) grains g^−1^ of spike dry weight at A + 7d for FE [32]. and 31.53 (E1_C18) to 38.84 g for the TGW. TGW had a higher broad-sense heritability of 0.75 compared to 0.25 for FE [32].

In Experiment II, ANOVA showed significant variation among different intra-spike partitioning traits (Table S2). Significant environmental effects were observed for all traits, and genotype-by-environment interaction effects were significantly different for most traits (Table S2). FE ranged from 36.19 to 46.46 grains g^−1^ (Table 1). Likewise, the glume partitioning index (GPI) varied from 0.21 to 0.24, the lemma partitioning index (LmPI) from 0.24 to 0.25, and the palea partitioning index (PPI) remained constant at 0.13. The awn partitioning index (API) ranged from 0.18 to 0.24 (Table 1). The sterile floret partitioning index (FPI) and the rachis partitioning index (RPI) ranged from 0.03 to 0.04 and 0.15 to 0.18, respectively. The broad-sense heritability for intra-spike partitioning traits was generally high, with the exception of FPI. API showed the highest heritability of 0.89, followed by GPI (0.88) and RPI (0.87). FE had an *H*^2^ value of 0.18 comparable to our association studies. These results are summarized in Table 1.

**Table 1:** Summary of phenotypic adjusted means and heritability (Experiment II)

Traits	E2_C18	E2_C19	E2_Q18	E2_Combined	H^2^
FE	36.19	46.46	45.46	40.88	0.18
GPI	0.22	0.21	0.24	0.22	0.88
LmPI	0.25	0.25	0.24	0.25	0.7
API	0.18	0.24	0.18	0.23	0.89
PPI	0.13	0.13	0.13	0.13	0.69
FPI	0.04	0.03	0.04	0.03	0.33
RPI	0.18	0.17	0.15	0.17	0.87

*FE* fruiting efficiency in grains g^−1^ of spike dry weight at anthesis + 7 days, *GPI* glume partitioning index, *LmPI* lemma Partitioning index, *API* awn partitioning index, *PPI* palea partitioning index, *FPI* floret partitioning index, *RPI* rachis partitioning index. E2_C18, E2_C19, E2_Q18, and E2_Combined refers to Citra 2018, Citra 2019, Quincy 2018 and combined dataset respectively for experiment II

### Phenotypic correlations among traits

FE showed significantly positive correlations with GY (*r* = 0.27** to 0.48***), GN (*r* = 0.47*** to 0.54**), and HI (*r* = 0.35** to 0.51***) [32], and negative correlation with TGW (*r* = −0.12 to 0.26**) (Table 2). Likewise, TGW showed significant positive correlations with HI (*r* = 0.15* to 0.26***) and GY (*r* = 0.21** to 0.24**), but negative correlation with GN (*r* = −0.31** to − 0.34**) and FE (−0.17* to −0.36**) (Table 2). Path coefficient analysis showed both FE and TGW had significant positive effects on HI (Table 3). Furthermore, FE had a positive indirect effect on HI through GN whereas TGW had negative indirect effects on GN.

**Table 2:** Pearson’s correlation coefficient between phenotypic traits using Best Linear Unbiased Estimates (Experiment I)

	Traits	HI	GY	GN	TGW
E1_C18	TGW	0.26**	0.24**	−0.34**	1
	FE	0.51***	0.27**	0.52***	−0.36**
E1_Q17	TGW	0.15*	0.03	−0.31**	1
	FE	0.48***	0.48***	0.54***	−0.23**
E1_Q18	TGW	−0.08	−0.04	−0.08	1
	FE	0.47***	0.38**	0.47***	−0.17*
E1_Combined	TGW	0.10	0.21**	−0.07	1
	FE	0.27**	0.36**	0.43***	−0.12

*HI* harvest index, *GY* grain yield in kg ha^−1^, *GN* Grain number m^−2^, *FE* fruiting efficiency in grains g^−1^ of spike dry weight at anthesis + 7 days, *TGW* thousand grain weight in g. E1_C18, E1_Q17, E1_Q18, and E1_Combined refers to Citra 2018, Quincy 2017, Quincy 2018 and combined dataset respectively for the experiment I. *, **, *** denotes significant at 0.05, 0.01 and 0.001 significance levels, respectively

**Table 3:** Direct and indirect effects on HI identified through path coefficient analysis (Experiment I)

E1_C18				
Traits	Direct effect	GN	FE	TGW
GN	0.434***	0.434	0.257	−0.199
FE	0.494***	0.226	0.494	−0.216
TGW	0.595***	−0.145	−0.150	0.595
E1_Q17				
Traits	Direct effect	GN	FE	TGW
GN	0.426***	0.426	0.186	−0.115
FE	0.341***	0.232	0.341	−0.090
TGW	0.376***	−0.130	−0.082	0.376
E1_Q18				
Traits	Direct effect	GN	FE	TGW
GN	0.336***	0.336	0.148	0.000
FE	0.31***	0.159	0.317	0.00
TGW	0.001	−0.026	−0.054	0.001
E1_Combined				
Traits	Direct effect	GN	FE	TGW
GN	0.353***	0.353	0.101	−0.030
FE	0.215***	0.165	0.215	−0.030
TGW	0.160**	−0.066	−0.040	0.160

*HI* harvest index, *GN* Grain number m^−2^, *FE* fruiting efficiency in grains g^−1^ of spike dry weight at anthesis + 7 days, *TGW* thousand grain weight in g. E1_C18, E1_Q17, E1_Q18, E1_Combined refers to Citra 2018, Quincy 2017, Quincy 2018 and combined dataset respectively for experiment I. *, **, *** denotes significant at 0.05, 0.01 and 0.001 significance levels, respectively

**Fig. 2:**
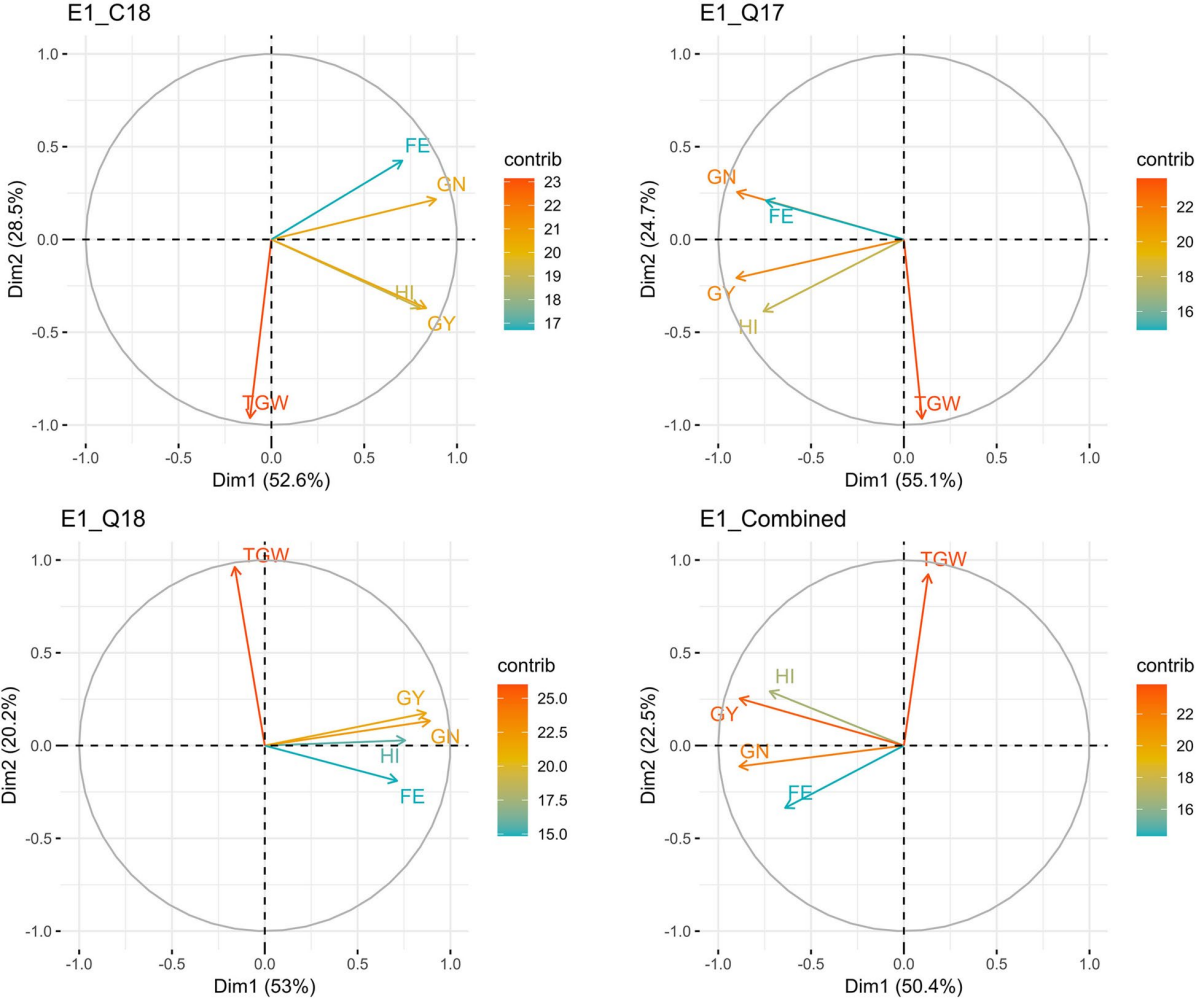
Principal component analysis biplot of measured traits using best linear unbiased estimates (BLUEs). HI, harvest index; GY, grain yield; GN, Grain number m^−2^; FE, fruiting efficiency; TGW, thousand grain weight. E1_C18, E1_Q17, E1_Q18, E1_Combined refers to Citra 2018, Quincy 2017, Quincy 2018, and combined dataset respectively for experiment I

FE also had negative correlations with various intra-spike portioning traits including GPI (*r* = − 0.25 to − 0.73*), API (*r* = − 0.16 to − 0.64*), FPI (*r* = −0.08 to − 0.75*), and RPI (*r* = −0.15 to −0.44) (Fig. 2). GPI showed positive correlations with FPI (*r* = 0.02 to 0.75*) and RPI (*r* = 0.29 to 0.82*). Likewise, LmPI had positive correlations with RPI (*r* = 0.01 to 0.30). Path coefficient analysis indicated that GPI, API, and LmPI had negative direct effects on FE in all datasets, except for E2_C18. In contrast, PPI had positive direct effects on FE (Table S3), while FPI showed negative direct effects on FE in all datasets, except for E2_Combined.

### PCA biplot analysis

**Fig. 3:**
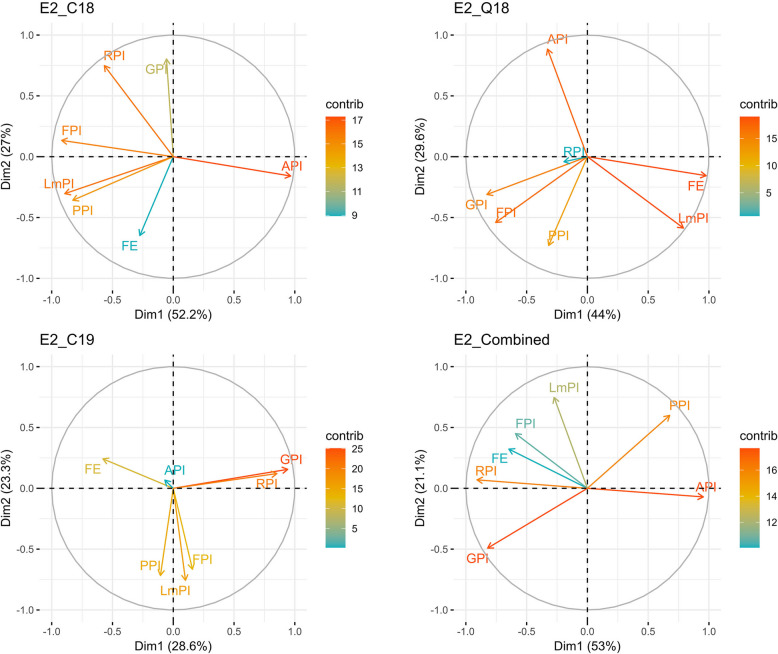
Principal component analysis biplot of measured traits using best linear unbiased estimates (BLUEs). FE, fruiting efficiency; GPI, glume partitioning index; LmPI, lemma Partitioning index; API, awn partitioning index; PPI, palea partitioning index; FPI, floret partitioning index; RPI, rachis partitioning index. E2_C18, E2_C19, E2_Q18, and E2_Combined refers to Citra 2018, Citra 2019, Quincy 2018 and combined dataset respectively for experiment II

**Fig. 4:**
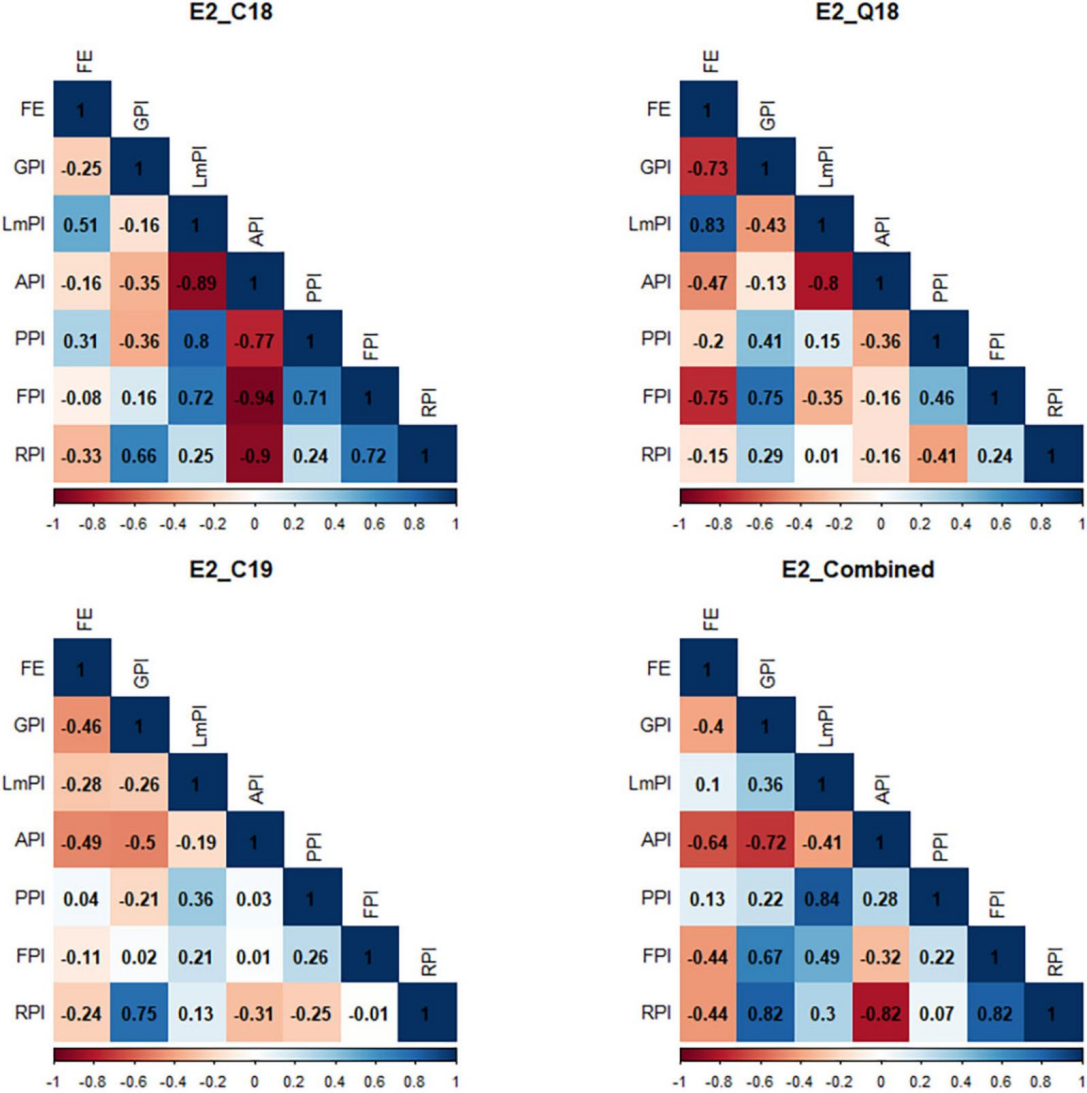
Pearson’s correlation coefficient between phenotypic traits using best linear unbiased estimates (Experiment II). FE, fruiting efficiency in grains g^−1^ of spike dry weight at anthesis + 7 days; GPI, glume partitioning index; LmPI, lemma Partitioning index; API, awn partitioning index; PPI, palea partitioning index; FPI, floret partitioning index; RPI, rachis partitioning index. E2_C18, E2_C19, E2_Q18, E2_Combined refers to Citra 2018, Citra 2019, Quincy 2018 and combined dataset respectively for the experiment II

The principal component analysis (PCA) biplot further supported the association between FE and other traits (HI, GN, TGW, API, FPI, GPI, RPI) from correlation analysis. In the first PCA, HI was close to GY, and FE was close to GN. The first two PCs explained 50.4 to 55.1% and 20.2 to 28.5% of the variation, respectively (Fig. 3). For intra-spike partitioning traits, the first two PCs explained 28.6 to 53% and 21.1 to 29.6% of the variation, respectively (Fig. 4). Furthermore, FPI, GPI, and RPI were clustered together, away from a cluster of LmPI and PPI whereas FE and API didn’t have any cluster.

### Genome-wide association study and gene annotation

Population structure and LD decay analysis using 20,706 SNPs were performed earlier [33]. To summarize, a total of 7,935 (38.32%), 7,496 (36.20%), and 5,275 (25.48%) SNPs were found in A, B, and D genomes, respectively. The PC analysis showed an admixture among genotypes with three clusters. The LD decay was found to be 3.4 Mbp for whole genome.

**Fig. 5:**
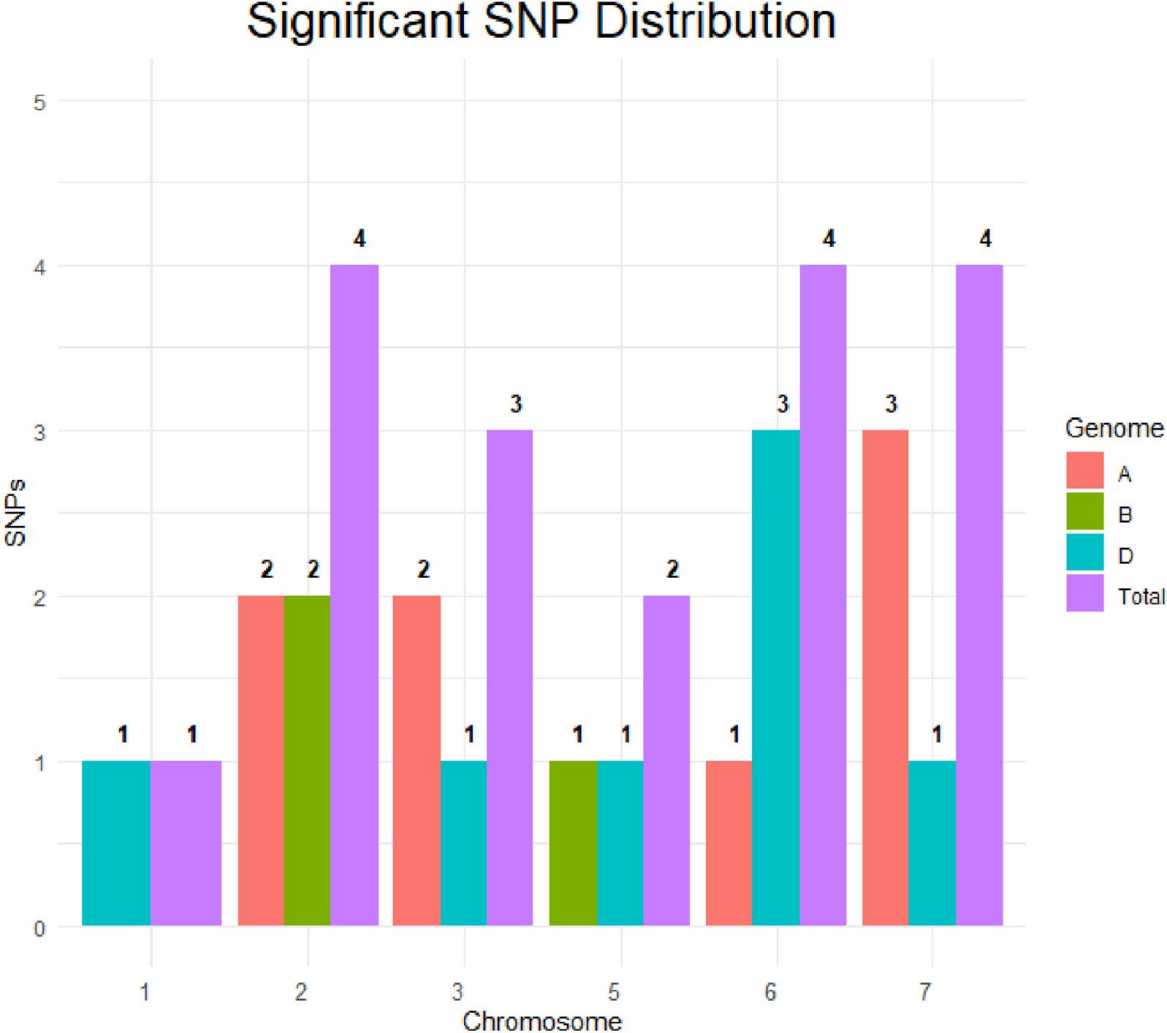
Marker-trait associations across three genomes in US soft wheat association panel

**Fig. 6:**
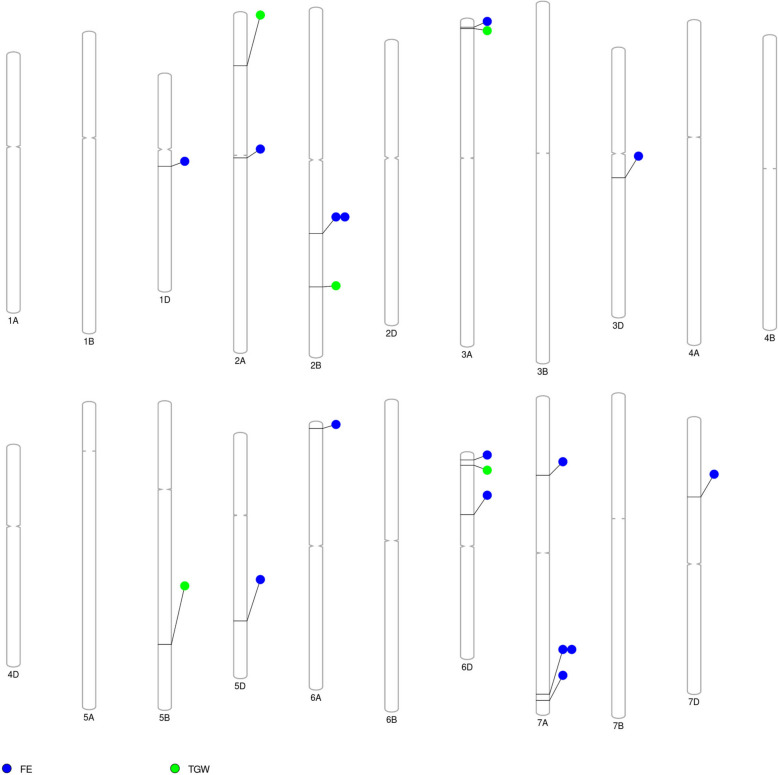
Overview of significant markers trait associations identified on each chromosome for phenotypic traits obtained from GWAS. FE, fruiting efficiency; TGW, thousand grain weight

Thirteen MTAs for FE were identified on 10 chromosomes (1D, 2A, 2B, 3A, 3D, 5D, 6A, 6D, 7A, and 7D) (Fig. 5, Fig. 6, Figure S1a) with phenotypic variation explained (PVE) ranging from 10.34 to 17.56% (Table 4). Additionally, 5 MTAs on chromosomes 2A, 2B, 3A, 5B, and 6D (Fig. 5, Fig. 6, Figure S1b) were significant with PVE ranging from 9.62 to 14.92% for TGW (Table 4). Two MTAs for FE were repeated in two environments.

**Table 4:** Summary of significant marker-trait associations for FE and TGW

Traits	SNPs	Chromosomes	PVE
FE	13	1A, 2A, 2B, 3A, 3D, 5D, 6A, 6D, 7A, 7D	10.34–17.56
TGW	5	2A, 2B, 5B, 6D	9.62–14.92

*FE* fruiting efficiency, *TGW* thousand grain weight

Functional gene annotation of the significant MTAs in the IWGSC RefSeq v1.0 reference genome identified several putative candidate genes, and their physical locations. Several MTAs were within genes with different biological and metabolic functions including zinc finger, F-box protein, nuclear pore complex protein, Oligopeptide transporter, and Fasciclin-like arabinogalactan protein (Table S4). Likewise, five genes that are in proximity (within 200 kb) of significant SNPs for FE were also found (Table S5).

### KASP marker validation

**Fig. 7:**
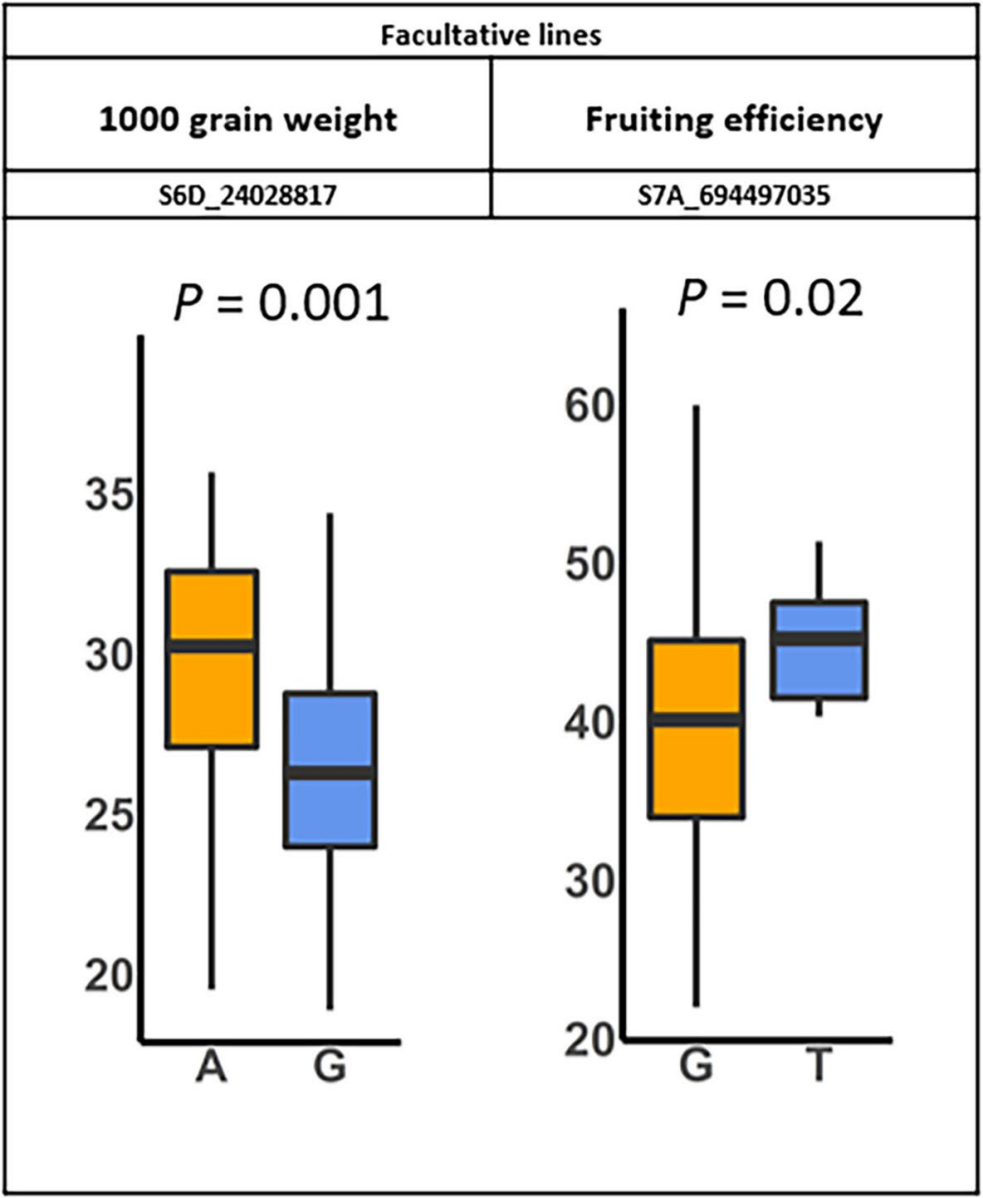
Boxplot representing KASP markers showed significant allelic effect in the diversity population. The KASP markers were identified using a studentized t-test (*P*-value < 0.05) in validation panel

A total of 7 KASP markers were developed and used for validation. Of these, one each newly developed KASP markers showed significant mean difference in FE and TGW traits, respectively, between two allelic groups in a validation population (Fig. 7 and Table S6). The VEP search in Ensembl showed that the two KASP markers were associated with un-identified protein or intergenic sequences. The KASP markers for both TGW and FE also showed significant phenotypic mean difference in other traits, particularly the TGW marker showed significant phenotypic mean difference in grain yield (Table S6).

## Discussion

The yield increase in the ‘Green Revolution’ era was mostly owing to an increase in HI; however, there has been little progress in improving HI in recent decades. The HI of currently used wheat cultivars remains at about 0.45 to 0.51 in spring wheat and 0.5–0.55 in winter wheat, which is lower than the theoretical upper limit of 0.62 [9, 34, 35]; therefore, there is still scope for improvement of HI in wheat and its stabilization across seasons and environments. Some recent studies have demonstrated that increased biomass had a positive association with grain yield, but a negative association with HI [34, 36]. To improve HI through converting increased biomass towards grain yield, it is essential to study novel partitioning traits that enable discrimination between “useful” and “non-useful” biomass [5]. The number of grains (sink strength) is a major trait for GY and HI improvement. There is strong evidence that during grain filling, wheat yield (grain weight per spike and spikes per unit area) is sink limited because carbon accumulation is limited by the total storage capacity of the grains. Therefore, improving the grain number per unit area is an important target in the genetic improvement of HI and yield potential. Additionally, grain number has more plasticity than grain weight and, therefore, is more responsive to genetic and environmental changes than grain weight [15, 37]. Understanding the genetic mechanisms of component traits that control grain number is pertinent for further improvement of GY and HI. Fruiting efficiency has been proposed as one of the novel promising traits that could be exploited to further increase the GN and HI [24].

The current study found a significant variation of FE in the US facultative soft wheat, which indicated the scope for improvement of this particular trait through breeding. This result agrees with several other studies [7, 19, 24, 38, 39]. Positive associations of wheat FE with GN, HI, and GY which have been observed in the current study and other recent studies [19, 24] were supported by PCA analysis that clustered these traits together. The positive physiological relationship between GN and FE has been reported [7, 8, 15, 24], with fruiting efficiency measuring the efficiency with which resources are used to set grains [7]. Higher FE indicates a declined abortion rate and increased survival of floret primordia during the stem elongation phase before anthesis [7, 15, 24], which eventually increases the likelihood of fertile florets to set grains [15, 37]. The low heritability of FE in our study suggested the complex nature and high environmental influence. The heritability estimate can be improved by including more replications, consistent environments, and precise measurement. However, various studies have demonstrated reasonable heritability [39–41] and a positive response to the selection of the FE trait [7]. Additionally, positive transgressive segregation reported for FE [7, 8, 39] suggested its applicability in further improvement of GN through selecting genotypes for higher FE. Previous studies have demonstrated that HI could be improved by (a) increasing SPI; and (b) reducing dry matter partitioning towards competing plant parts specifically internode 2 and/or internode 3 [20]. Reducing second or third internode length to increase HI might have to involve height reduction, however, further reducing already optimum wheat plant height (0.7–1 m) may penalize wheat yield [15, 16]. This could limit the extensive use of stem partitioning traits up to a specific point. Thus, improving FE could be a pragmatic way to increase GN and HI through the improvement of the spike partitioning index and the internode partitioning index in wheat. Pyramiding QTLs linked to these partitioning traits [33] in high FE genotypes could further boost harvest index and grain yield.

Utilization of FE in the breeding program may need to consider two potential trade-offs: spike weight at anthesis and grain weight [24, 39]. A negative association has been reported between FE and SPI in some studies [7, 18, 26, 27]. The negative correlation between SPI and FE suggests that selection for high FE may reduce SPI. However, different authors suggested the possibility of identifying genotypes with high SPI and FE [5, 7, 19, 20]. Marker-assisted selection may facilitate combining the genes that control these traits. In the current study, a negative relationship between TGW and FE suggests an increase in FE resulted in a decrease in grain weight, consistent with most previous studies [15, 16, 19, 24, 28, 39] except that González et al. (2014) [22] did not find such an association. A clear trade-off occurs only if grain weight at each floral position is reduced as a result of an increase in FE, which would reduce GY [7, 42]. However, some studies suggested that the reduction in grain weight owing to increased FE usually occurred at distal smaller florets that were infertile in low FE genotypes and the proximal florets remained unaffected, thereby the reduction in the average grain weight is not a trade-off, but due to increased fertile florets [7, 10, 15, 39]. Improving vascular connections within the rachilla can reduce resistance and improve floret fertility of more distal florets, therefore, minimizing such trade-offs [36, 43].

Among 13 MTAs for FE, five MTAs were in genes (Table 4, Table S4). These putative candidate genes, along with nearby genes (Table S5), can be used to develop selectable markers for further improvement of FE. Since the heritability of FE was found to be relatively low, marker-assisted selection would play an important role in the improvement of this trait. Out of 5 MTAs identified for TGW, 4 MTAs were found within genes (Table 4, Table S4). Previous studies identified QTLs/MTAs responsible for TGW on 2A [44–46], 2B [33, 46], 3A [33, 47, 48], and 6D [46]. An MTA S6D_24028817 was in a gene *TraesCS6D02G049500* which is annotated as an Oligopeptide transporter protein (Table S4). Oligopeptide transporters (OPTs) are membrane-localized proteins with various biological processes, such as substrate transportation [49]. An MTA S2B_644162831 found within gene *TraesCS2B02G450900* was annotated as a Nucleoporin protein (Table S4). Nucleoporin proteins have different functions such as nucleocytoplasmic transport, cell differentiation, cell signaling, and gene expression [50–52]. Likewise, an MTA S6D_11346685 was found in a gene *TraesCS1B02G401200* which annotates for the F-box family protein. This protein plays a role in the developmental and physiological processes such as spike development, and pollen recognition in plants [53, 54]. These genes, along with those in close proximity to significant SNPs (Table S5) and their associated functions denote their potential for further manipulation to improve TGW. In addition, we found significant SNPs associated with FE which had an allelic effect in the same direction on other traits along with FE.

Negative associations were observed between FE and intra-spike partitioning traits including awn, glume, sterile floret, and rachis partitioning. These non-grain sinks compete with florets for assimilates, resulting in lower FE. The negative relation between RPI and FE could be taken as an example of such competition. Both GPI and sterile FPI have a significant negative correlation with FE. Although the physiological basis for that relationship is not clear, high negative indirect effects through rachis PI seem to be a driving force in both cases. The highly negative direct effect of awn PI on FE shows that the formation of awns and fertile florets competes for the same source of assimilates in a spike, and investment of dry mater on awns ultimately reduces FE. Awns can increase spike surface area by up to 50% [55], and contribute to total photosynthesis [56, 57]; whereas awnletted wheat may produce significantly more grains, therefore, awn elimination could potentially enhance grain numbers [24, 58–60]. Since grain yield in wheat is mostly sink limited during grain filling, photosynthetic contribution by awns towards grain yield/number might not be enough to counterbalance the extent of assimilate investment towards their production and physiological maintenance.

Higher FE could be achieved through a preferential allocation of spike DM to the florets with a proportional reduction in partitioning to non-productive sinks or other structural parts (rachis, glumes, awns). It can be assumed that competition for assimilates leads to the shortage of assimilates, resulting in floret primordia abortion [61]. Higher availability of assimilates to florets improves the probability to turn labile floret primordia into fertile florets, which results in increased FE and GN [24]. In addition, increasing spike sugar content [19, 27, 62] and modifying plant signaling responses [17, 19, 20] also increase FE and GN. In summary, FE can be increased through decreasing intra-spike dry matter partitioning to awn, rachis, and glume, and potentially increasing partitioning to the lemma and palea within the spikelet morphological components. This optimized partitioning would eventually have a positive impact in reaching the theoretical HI limit of above 60%. Two KASP markers, one significantly contributing to TGW and the other to FE, could be used for future breeding programs intended to select elite lines for grain partitioning traits. Additional research is needed to fine mapping region encompassing these KASP markers to determine the underlying genes and their subsequent functions.

## Conclusions

Results showed that FE is strongly associated with GN, HI, and grain yield. Increasing FE would most likely contribute to higher GN. Reducing glume, awn, and rachis partitioning and increasing partitioning of lemma and palea would further increase FE and GN. GWAS analysis suggested that the genetic control of FE is complex and controlled by several putative QTL regions. GWAS analysis successfully identified 18 markers significantly associated with FE and TGW. Nine MTAs for FE and TGW were identified to be within genes which are potential targets of selection in marker-assisted breeding to capitalize genetic variation for the trait. KASP marker development and validation would facilitate transfer of these QTL into adapted cultivars through a marker-assisted breeding to improve GY and HI.

## Methods

### Trait evaluation in field experiments

Three experiments were conducted in two locations in northern and north-central Florida. The genetic materials and environments involved in the first experiment (Experiment I) have been described in Shahi et. al (2022) [32]. In brief, a panel of 236 facultative soft wheat elite lines and varieties were planted in the Plant Science Research and Education Unit (PSREU) in Citra, Florida in 2017–2018, and in the North Florida Research and Education Center (NFREC) in Quincy in 2016–2017 and 2017–2018 using an augmented design with three repeated checks, SS8641 (PI 674197), AGS2000 (PI 656845), and Jamestown (PI 653731) to control spatial variability. These check cultivars are popular commercial soft wheat varieties grown throughout the southern and south-eastern US. The checks were replicated twice in each block. The weather conditions of experiments are provided in Table S7.

Measurement of traits HI, GY, GN, and FE has already been described in Shahi et al. (2022) [32]. Days to anthesis was taken for each plot as the days from planting to when 50% of plants flowered [63]. At 7 days after anthesis (A + 7d, Zadoks scale: GS70), wheat tillers were cut at ground level from a 0.25 m^2^ area of each plot. The sample was oven-dried at 60 °C for 72 h and then dried biomass was weighted and converted to g m^−2^. Spikes were separated from stem and leaves, weighed, and converted to spike dry matter m^−2^ at A + 7d. Traits such as GN, GY, TGW, and HI were recorded at physiological maturity (Zadoks scale: GS90). Days to physiological maturity were taken for each plot when 50% of the peduncle turned yellow. At physiological maturity, plants from a 0.25 m^2^ plot area were harvested and threshed. Grain in each plot was weighed and converted to g m^−2^. Thousand-grain weight (TGW) was obtained by weighing 1,000 kernels counted in a seed counter (Seedburo Equipment Co., Chicago, IL). GN was calculated by dividing grain yield m^−2^ by mean grain weight (TGW/1000). HI was measured as the ratio of grain yield m^−2^ to above-ground dry biomass m^−2^. Likewise, GY was measured in kg ha^−1^ as the total grain weight per plot divided by the plot area adjusted to 12% moisture. FE was calculated as a ratio of GN m^−2^ at maturity to spike dry weight m^−2^ at A + 7d and expressed as grain numbers per gram of spike dry weight at A + 7d.

A sub-set of 10 genotypes from the panel used in Experiment I were evaluated for non-grain spike partitioning (Experiment II) at PSREU, Citra, Florida in the 2017–2018 and 2018–2019 growing seasons, and at NFREC, Quincy, Florida in 2017–2018 growing season (similar seed rate and plot size). The experiments used a randomized complete block design (RCBD) with 3 replications and the same agronomic and management practices as described in Experiment I. Seven spikes were randomly selected from each plot at harvest and separated into different spike parts (awns, rachis, glume, lemma, palea, and infertile floret). The dry weight (DW) of total spikes and each of the spike parts and grains were also measured to calculate the partitioning of each part. The awn partitioning index (API) was calculated as (DW of awns)/ (DW of total spike—grain DW). Similarly, rachis, glume, lemma, palea, and infertile floret partitioning indices were calculated as the DW of the respective spike part divided by the DW of the non-grain spike calculated as the difference of total spike DW and grain DW.

A validation panel consisting of two diversity populations of 178 facultative lines and 59 spring lines was planted during 2018–2019 season to validate KASP markers (Experiment III). The populations were planted at the Plant Science Research and Education Unit (PSREU) in Citra, Florida. A randomized augmented design with three repeated checks (“AGS 2000”, “SS8641”, and “Jamestown”) was used for the experiment.

### Genotyping and KASP marker development

Detailed description of the methodologies for genotyping have been provided previously [32]. In brief, high-quality DNA was extracted from freeze-dried, powdered leaf tissue obtained from 2-week-old plants using modified CTAB (cetyltrimethylammonium bromide) protocol and genotyping-by-sequencing (GBS) library was constructed using two restriction enzymes, *Msp*I and *Pst*I-HF [64]. SNPs were called using the TASSEL v5.0 GBS v2.0 discovery pipeline [65] and the IWGSC RefSeq v1.1 reference genome [66].

Significant SNPs identified from GWAS were selected for the development of KASP markers. Primers for KASP were designed based on the sequences flanking the SNPs in the Chinese Spring reference genome RefSeq v1.0 (IWGSC 2018) using PolyMarker (http://www.polymarker.info/), which considers the polyploid nature of multiple homologs. Designed primer sequence was searched in entire genome to remove possible duplication and only the markers that showed clear cluster separation between two alleles were selected. The KASP assays were carried out in a GeneAmp™ PCR System 9700 Fast Thermal Cycler at USDA-ARS Central Small Grain Genotyping Lab, Manhattan, Kansas. A 5 µL of KASP PCR mix composed of 1.94 µL of 2 × PACE Genotyping Master Mix (https://3crbio.com/), 0.06 µL primer mix, and 3 µL DNA at 10–20 ng/µl. Amplification of PCR began with a denaturation of 94 °C for 15 min, followed by 10 touch-down PCR cycles at 94 °C for 20 s and 67 °C for 60 s with −1.0 ^0^C/cycle, and then 32 cycles at 94 °C for 20 s and 57 °C for 60 s.

### Phenotypic data analysis

Analysis of variance (ANOVA) was performed using the “lme4” package [67] in R software (v3.5.1, R Development Core Team). Best linear unbiased estimates (BLUEs) were calculated for each environment separately as well as across all environments. In the analysis, the genotypic effects were considered fixed, while all the other effects were regarded as random. All traits were adjusted using days to anthesis as a covariate. The following model applied to individual environments for experiment I and III.

$${\text{Y}}_{\text{ijkl}}=\upmu +{\text{B}}_{\text{i}}+{\text{ID}}_{\text{j}}+{\text{G}}_{\text{k}}+{\text{C}}_{\text{l}}+{\upvarepsilon }_{\text{ijkl}}$$

For combined data across environments, the following model was used.

$${Y}_{\text{ijk}}=\upmu +\text{I}+\text{G}+\text{C}+{E}_{\text{i }}+ {I\times E}_{\text{i }}{+{G\times E}_{\text{i }}+{C\times E}_{\text{i }}+\text{B}}_{k}{(E}_{\text{i }})+{\upvarepsilon }_{\text{ijk}}$$

where Y is the trait of interest; μ is the effect of the mean; G corresponds to the un-replicated genotypes; C is the effect of the replicated checks on each block; E_i_ is the effect of the *i*th environment, and I is the effect of the identifier of the checks. I × E_i_, G × E_i_, and C × E_i_ are the effects of the check identifier by environment, genotype by environment, and check by environment interactions, respectively; B_k_(E_i_) is the block effect nested within each environment; and ε is the standard normal error [68].

To estimate BLUEs for experiment II (RCBD), the following model was used for individual environments.

$${Y}_{\text{ijk}}=\upmu ++\text{Gj }{+\text{B}}_{\text{k}}+{\upvarepsilon }_{\text{ijk}}$$

The following model was used for combined environments.

$${Y}_{\text{ijk}}=\upmu +{\text{E}}_{\text{i }}+\text{Gj }{+{\text{G}\times \text{E}}_{\text{i }}+\text{B}}_{\text{k}}{(\text{E}}_{\text{i }})+{\upvarepsilon }_{\text{ijk}}$$

where the phenotypic response (Y_ijk_) is a function of the overall mean (µ), j^th^ genotype (G_i_), i^th^ environment, genotype-environment interaction (${\text{G}\times \text{E}}_{\text{i}}$), B_k_(E_i_) is the block effect nested within each environment, and ε is the standard normal error.

Broad-sense heritability was calculated using the following formula:

$${H}^{2}=\frac{{\upsigma }^{2}\text{G}}{{\upsigma }^{2}\text{G}+\frac{{{\upsigma }^{2}}_{\text{G}\times \text{E}}}{\text{n}}+\frac{{\upsigma }^{2}\text{e}}{\text{nr}}}$$

where *H*^2^ is the broad-sense heritability estimate, ${\upsigma }^{2}\text{G}$ is the genetic variance, ${{\upsigma }^{2}}_{\text{GXE}}$ is the genotype by environmental variance, ${\upsigma }^{2}\text{e}$ is the residual variance, n is the number of environments, and r is the number of replications.

Pearson’s correlation among traits was computed using the “corrplot” package in R [69]. A PCA biplot was created with the “factoextra” package in a R [70]. Additionally, path coefficient analysis was conducted in R using the “lavaan” package [71]. In the first analysis, GN, FE, and TGW were used as predictors whereas HI was designated as a response. To estimate the direct and indirect effects of different traits on FE, glume partitioning index (GPI), lemma partitioning index (LmPI), awn partitioning index (API), palea partitioning index (PPI), floret partitioning index (FPI), and rachis partitioning index (RPI) were designated as predictors whereas FE was designated as a response.

### Genome-wide association study and gene annotation

The compressed mixed linear model (CMLM) of the genome association predicted integrated tool (GAPIT) in R [72] was used to identify the associations between phenotypic and genotypic data. A GWAS was performed using adjusted means (BLUEs) from the dataset collected from Citra (2017–2018) [E1_C18], Quincy (2016–2017) [E1_Q17] and Quincy (2017–2018) [E1_Q18], and combined data (E1_Combined) in Experiment I. Significant MTAs were claimed at a threshold of FDR (*p* < 0.10). We used “cmplot” package in R to generate SNP density plots, Manhattan plots, and Q-Q plots. IWGSC wheat reference genome RefSeq v1.0 [65] was used to find candidate genes associated with significant MTAs. Their annotations were determined using the Variant Effect Predictor (VEP) tool in the Ensemble Plants (http://plants.ensembl.org/Triticum_aestivum/ Tools/VEP).

In the validation analysis, a student's t-test was utilized. However, if non-normal residuals were observed, the kruskal–wallis test was employed. Differences in means between line groups with two or combinations of allelic types were computed. A KASP marker was deemed effective if there was significant mean difference between the contrasting allelic groups.

## Supplementary Information

Additional file 1: Figure S1a Manhattan plot (left) and quantile-quantile plots (right) showing genome-wide SNP loci associated with fruiting efficiency (FE) ordered on C18, Q17, Q18, and Combined. The horizontal line in Manhattan plot represents the expected value with a uniform suggestive genome wide significance threshold [-FDR ≤ 0.10]. Figure S1b Manhattan plot (left) and quantile-quantile plots (right) showing genome-wide SNP loci associated with Thousand Grain Weight (TGW) ordered on C18, Q17, Q18, and Combined. The horizontal line in Manhattan plot represents the expected value with a uniform suggestive genome wide significance threshold [-FDR ≤ 0.10].

Additional File 2: Table S1 Summary of ANOVA results testing the effects of genotype (G), environment (E), and genotype-by-environment interaction (G×E) in phenotypic traits evaluated in combined analysis of experiment I. The table includes mean square values and significance level of each term. Table S2 Summary of ANOVA results testing the effects of genotype (G), environment (E), and genotype-by-environment interaction (G×E) in phenotypic traits evaluated in combined analysis of experiment II. The table includes mean square values and significance level of each term. Table S3 Direct and indirect effects of different traits on fruiting efficiency (FE) identified through path coefficient analysis (Experiment II). Table S4 Summary of all significant markers and their functional annotations associated with two traits. Table S5 Information on genes at proximity to significant SNPs for phenotypic traits. Table S6 Significant KASP markers detected in validation study (previously identified by an association mapping study). Table S7 Weather table showing Tave (monthly average temperature) and Ppt (monthly precipitation in mm). Association mapping panel was planted two seasons in Citra (2017/ 2018) and Quincy (2016/2017, 2017/2018).

## Data Availability

The datasets and R code used during the current study are available in the https://doi.org/10.6084/m9.figshare.26046508.v1.

## Abbreviations

ANOVA: Analysis of Variance
API: Awn partitioning index
BLUE: Best Linear Unbiased Estimation
CMLM: Compressed Mixed Linear Model
FPI: Floret partitioning index
FE: Fruiting efficiency
GAPIT: Genome Association Predicted Integrated Tool
GWAS: Genome -Wide Association Studies
GPI: Glume partitioning index
GN: Grain Number
GY: Grain Yield
HI: Harvest Index
KASP: Kompetitive allele specific PCR
LmPI: Lemma Partitioning index
LD: Linkage Disequilibrium
MTA: Marker-Trait Association
PPI: Palea partitioning index
PVE: Percent Variation Explained
QTL: Quantitative Trait Loci
RPI: Rachis partitioning index
SPI: Spike Partitioning Index
TGW: Thousand grain weight
