# Supplementary material for: Dissecting the genetic basis of fruiting efficiency for genetic enhancement of harvest index, grain number, and yield in wheat
Source: BMC Plant Biol. 2025 Jan 24;25:101. doi: 10.1186/s12870-025-06072-1 (PMC11760100; doi:10.1186/s12870-025-06072-1)
Supplement: Supplementary file 1 — Additional file 1: Figure S1a Manhattan plot (left) and quantile-quantile plots (right) showing genome-wide SNP loci associated with fruiting efficiency (FE) ordered on C18, Q17, Q18, and Combined. The horizontal line in Manhattan plot represents the expected value with a uniform suggestive genome wide significance threshold [-FDR ≤ 0.10]. Figure S1b Manhattan plot (left) and quantile-quantile plots (right) showing genome-wide SNP loci associated with Thousand Grain Weight (TGW) ordered on C18, Q17, Q18, and Combined. The horizontal line in Manhattan plot represents the expected value with a uniform suggestive genome wide significance threshold [-FDR ≤ 0.10]. [file 12870_2025_6072_MOESM1_ESM.docx]

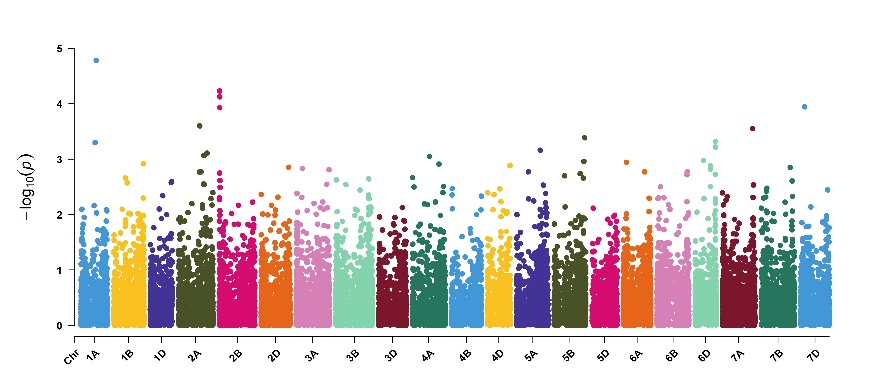

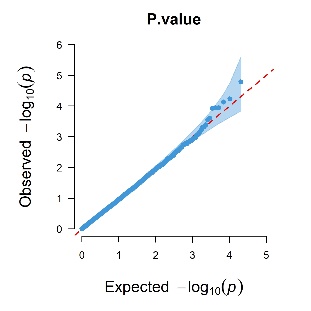


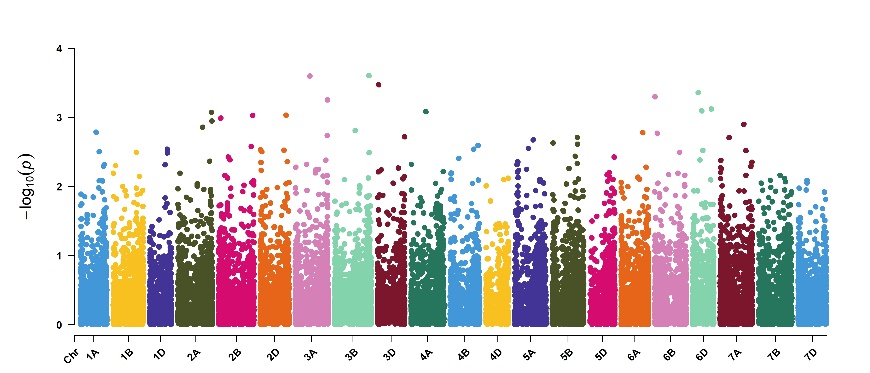

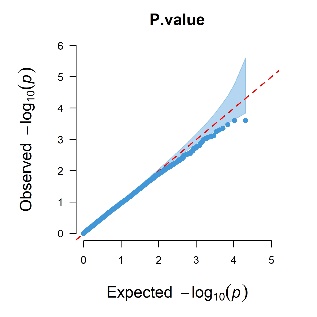


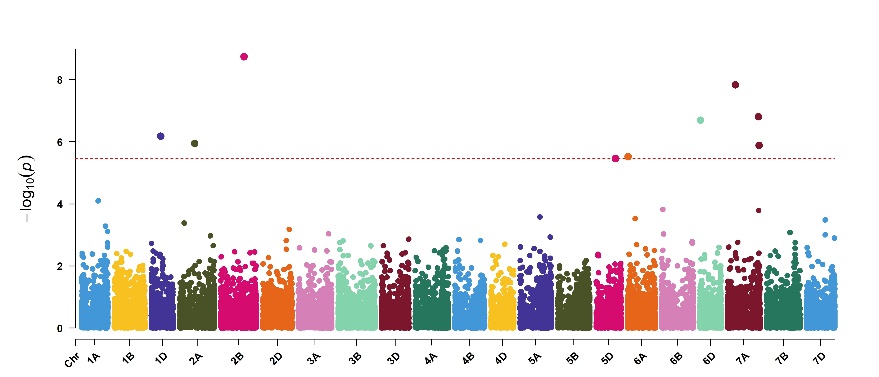

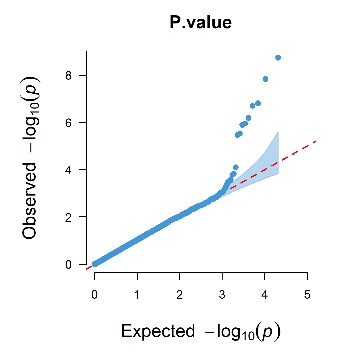


#
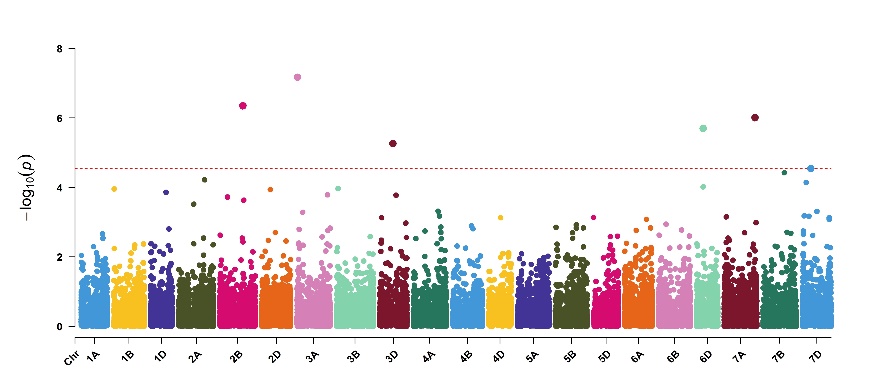

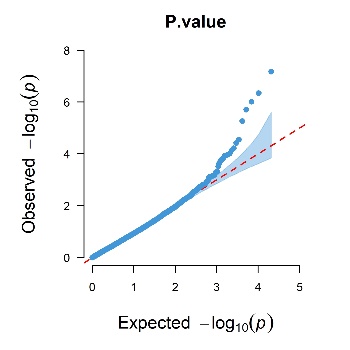


# Figure S1a. Manhattan plot (left) and quantile-quantile plots (right) showing genome-wide SNP loci associated with fruiting efficiency (FE) ordered on C18, Q17, Q18, and Combined. The horizontal line in Manhattan plot represents the expected value with a uniform suggestive genome wide significance threshold [-FDR ≤ 0.10].


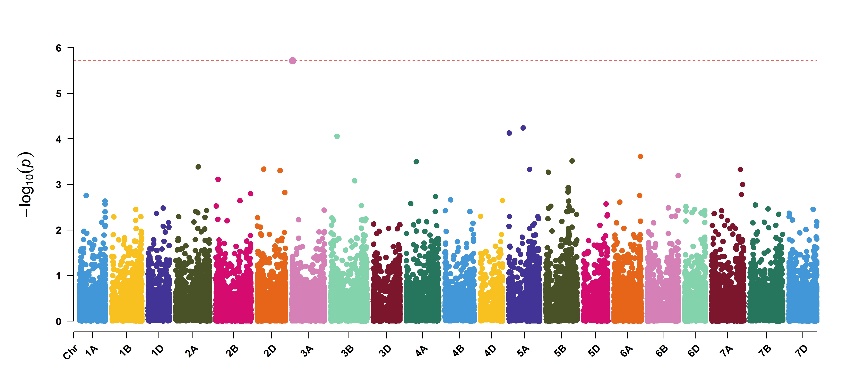

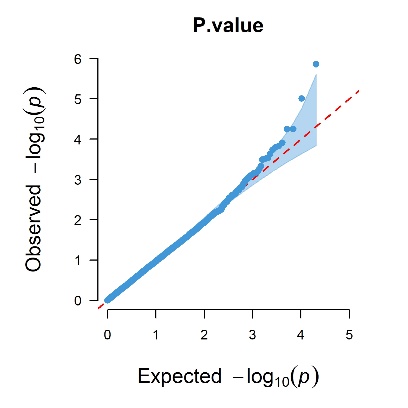


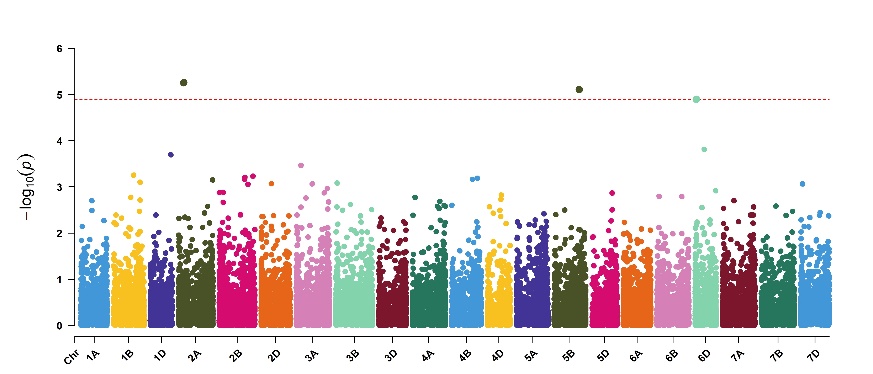

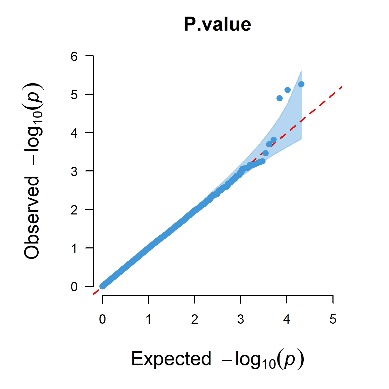


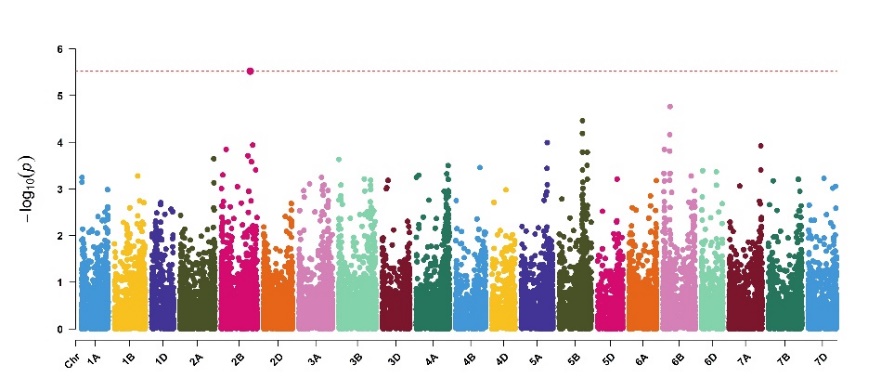

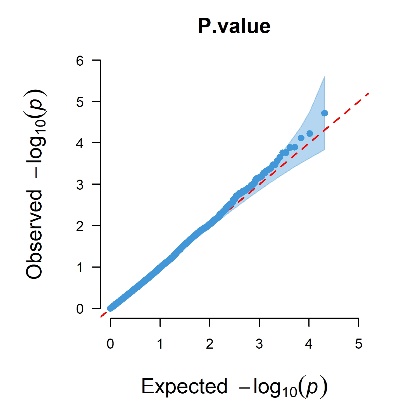


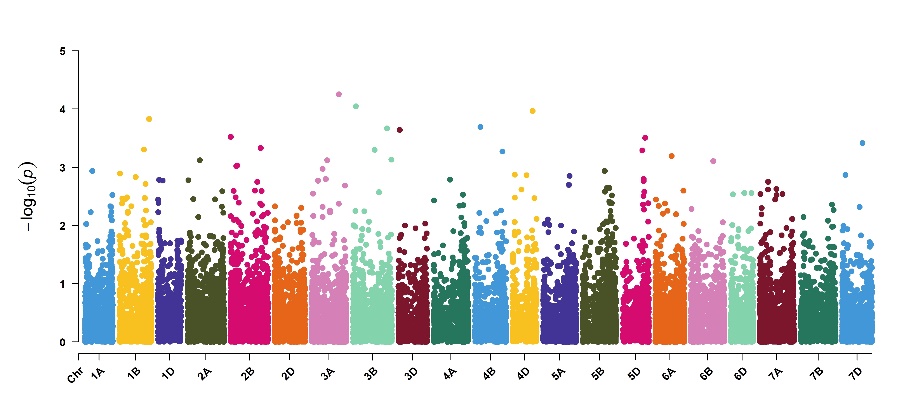

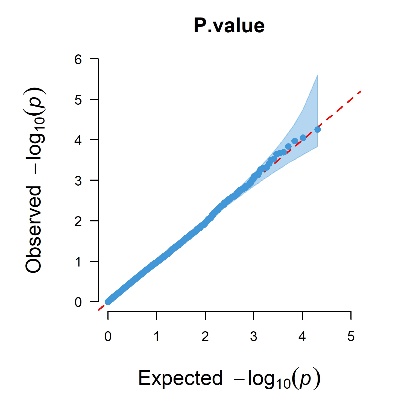


Figure S1b. Manhattan plot (left) and quantile-quantile plots (right) showing genome-wide SNP loci associated with Thousand Grain Weight (TGW) ordered on C18, Q17, Q18, and Combined. The horizontal line in Manhattan plot represents the expected value with a uniform suggestive genome wide significance threshold [-FDR ≤ 0.10].
